# Respiratory Health in Cleaners in Northern Europe: Is Susceptibility Established in Early Life?

**DOI:** 10.1371/journal.pone.0131959

**Published:** 2015-07-13

**Authors:** Øistein Svanes, Trude Duelien Skorge, Ane Johannessen, Randi Jacobsen Bertelsen, Magne Bråtveit, Bertil Forsberg, Thorarin Gislason, Mathias Holm, Christer Janson, Rain Jögi, Ferenc Macsali, Dan Norbäck, Ernst Reidar Omenaas, Francisco Gómez Real, Vivi Schlünssen, Torben Sigsgaard, Gunilla Wieslander, Jan-Paul Zock, Tor Aasen, Julia Dratva, Cecilie Svanes

**Affiliations:** 1 Department of Clinical Science, University of Bergen, Bergen, Norway; 2 Department of Thoracic Medicine, Haukeland University Hospital, Bergen, Norway; 3 Department of Occupational Medicine, Haukeland University Hospital, Bergen, Norway; 4 Centre for Clinical Research, Haukeland University Hospital, Bergen, Norway; 5 Department of Public Health and Primary Health Care, University of Bergen, Bergen, Norway; 6 Occupational and Environmental Medicine, Umeå University, Umeå, Sweden; 7 Department of Respiratory Medicine and Sleep, National University Hospital of Iceland, Reykjavik, Iceland; 8 Department of Occupational and Environmental Medicine, University of Gothenburg, Gothenburg, Sweden; 9 Department of Medical Sciences, Occupational and Environmental Medicine, University of Uppsala, Uppsala, Sweden; 10 Lung Clinic, Tartu University Hospital, Tartu, Estonia; 11 Department of Gynecology and Obstetrics, Haukeland University Hospital, Bergen, Norway; 12 Department of Public Health, Section for Environment, Occupation and Health, Aarhus University, Aarhus, Denmark; 13 Centre for Research in Environmental Epidemiology (CREAL), Barcelona, Spain; 14 Swiss Tropical and Public Health Institute (SwissTPH), University of Basel, Basel, Switzerland; 15 Centre for International Health, University of Bergen, Bergen, Norway; Haukeland University Hospital, NORWAY

## Abstract

**Rationale:**

There is some evidence that maternal smoking increases susceptibility to personal smoking’s detrimental effects. One might question whether early life disadvantage might influence susceptibility to occupational exposure.

**Objectives:**

In this cross-sectional study we investigated respiratory symptoms, asthma and self-reported chronic obstructive pulmonary disease (COPD) as related to working as a cleaner in Northern European populations, and whether early life factors influenced susceptibility to occupational cleaning’s unhealthy effects.

**Methods:**

The RHINE III questionnaire study assessed occupational cleaning in 13,499 participants. Associations with respiratory symptoms, asthma and self-reported COPD were analysed with multiple logistic regressions, adjusting for sex, age, smoking, educational level, parent´s educational level, BMI and participating centre. Interaction of occupational cleaning with early life disadvantage (maternal smoking, severe respiratory infection <5 years, born during winter months, maternal age at birth >35 years) was investigated.

**Main Results:**

Among 2138 ever-cleaners the risks of wheeze (OR 1.4, 95% CI 1.3–1.6), adult-onset asthma (1.5 [1.2–1.8]) and self-reported COPD (1.7 [1.3–2.2]) were increased. The risk increased with years in occupational cleaning (adult-onset asthma: ≤1 year 0.9 [0.7–1.3]; 1–4 years 1.5 [1.1–2.0]; ≥4 years 1.6 [1.2–2.1]). The association of wheeze with cleaning activity ≥4 years was significantly stronger for those with early life disadvantage than in those without (1.8 [1.5–2.3] vs. 1.3 [0.96–1.8]; p_interaction_ 0.035).

**Conclusions:**

Occupational cleaners had increased risk of asthma and self-reported COPD. Respiratory symptom risk was particularly increased in persons with factors suggestive of early life disadvantage. We hypothesize that early life disadvantage may increase airway vulnerability to harmful exposure from cleaning agents later in life.

## Introduction

Several studies have shown that cleaners have more airway symptoms and asthma than a general population [1, 2], but even though exposure to cleaning agents is a risk factor for asthma, not all cleaners develop such problems. Likewise, only a limited proportion of smokers develop COPD. An analysis of the European Community Respiratory Health Survey showed that early life disadvantage was associated not only with adult lung function level, but also with accelerated lung function decline [3]. Why early life development may influence lung ageing decades later is not well understood. Two recent analyses revealed that persons with characteristics reflecting early life disadvantage were more susceptible to the harmful effects of adult smoking: Guerra *et al* [4] found stronger effects on airflow limitation of personal smoking in those exposed to parental smoking. Stronger detrimental effects of smoking on lung function decline was found in persons exposed to maternal smoking, born during the winter season, with maternal age at birth above 35 years and/or severe childhood respiratory infections (Dratva et al., 2014, manuscript in preparation). In these analyses, early life disadvantage factors appeared to increase the vulnerability to adult active smoking. Smoking involves a complex mix of harmful chemical exposures and it would be reasonable to suspect a similar interaction for other harmful chemical exposures. We hypothesise that early life disadvantage factors may modify adult responses to occupational exposures, such as cleaning agents.

There is little knowledge on the risk of COPD in cleaners. Smoking is still the single most important cause of COPD, but as the prevalence of smoking declines, non-smoking factors such as working conditions may become increasingly more important [5]. It seems plausible that exposure to different types of airways irritants (gases and aerosols) from cleaning agents over time might lead to COPD development among cleaners, but as of today this is not well investigated [6, 7].

The Respiratory Health In Northern Europe (RHINE) study provided an opportunity to investigate cleaners in a large population-based sample that included information on early life exposures. In addition, the study has sufficiently large numbers to address effect modification. In this article we investigated associations between working as a cleaner and respiratory symptoms, asthma and self-reported COPD, taking the duration of exposure into account, and we investigated whether early life factors modified the adverse effects of occupational cleaning on respiratory health.

## Methods

### Design and subjects

From 1990 to 1994, randomly selected population-based samples of men and women aged 20 to 44 years were invited to a postal screening questionnaire as part of the European Community Respiratory Health Survey (ECRHS) I stage I. The RHINE study is a postal questionnaire follow-up study of the 21,659 persons who participated from the seven Nordic and Baltic centres (Bergen in Norway; Umeaa, Gothenburg and Uppsala in Sweden; Aarhus in Denmark; Reykjavik in Iceland and Tartu in Estonia). From 1999 to 2001, a comprehensive postal questionnaire was answered by 16,106 responders (75%). The timing corresponded with the second follow-up of ECRHS II and, thus, is referred to as the RHINE II study. The third stage of the RHINE study, RHINE III, took place from 2010 to 2012, and included 13,499 responders (62% of the original sample) [8]. The questionnaires in RHINE II and RHINE III included questions on respiratory symptoms, asthma, rhinitis, bronchitis, smoking, indoor environment, occupation, early life exposures and sleep disorders. This article presents data from 13,283 persons who responded to a question on having worked as a cleaner (included only in the RHINE III questionnaire).

Written consent was obtained from all the participants at each stage of the study. The Regional Committees for Medical and Health Research Ethics West in Norway, the National Bioethics Committee in Iceland, the Research Ethics Committee of the University of Tartu in Estonia, The Regional Ethical Review Board in Uppsala, Sweden and the Scientific Committees for Central Jutland in Denmark approved each stage of the study.

### Outcomes

The standard ECRHS questions were used to assess respiratory symptoms and diseases (for wording see www.ecrhs.org). Wheezing and other respiratory symptoms were reported as 12 months prevalence, while the respiratory diseases adult asthma and COPD were reported as lifetime prevalence. Asthma was defined on participants’ report of having or ever having had asthma; adult asthma was defined as onset of asthma after the age of 16 years. Self-reported COPD was defined by the question “Has a doctor ever told you that you have chronic obstructive pulmonary disease (COPD)?” Chronic bronchitis was defined as participants reporting bringing up phlegm almost every day for at least three months in two consecutive years [9, 10]. “Three or more asthma symptoms” was defined as having answered yes to three or more of the following symptoms in the last 12 months: Wheezing or whistling from the chest; breathless when wheezing; wheezing or whistling when not having a cold; woken with feeling of tightness in chest; been woken by attack of shortness of breath; been woken by attack of cough; attack of asthma; currently taking any asthma medicine [11].

### Exposure

“Occupational cleaner” and “years worked as an occupational cleaner” were defined from the questions “*Have you ever worked as a cleaner / cleaning assistant*?*”* and “*If yes*, *for how many years*?*”*. Participants who had ever worked as a cleaner were treated as exposed, whereas all other participants were treated as unexposed, thus, persons with other occupational exposures are included in the reference category. Based on the exposure duration in tertiles we built a categorical variable: never exposed, exposed for ≤1 year, >1 to <4 years, ≥4 years.

### Covariates

Body mass index was calculated from self-reported weight and height, as weight in kilos per square height in meters. Smoking history was assessed by the questions *“Are you a smoker*?*”* and *“Are you an ex-smoker*?*”*; defining never smokers, current smokers and ex-smokers. Participants’ and their parents’ educational level (primary school, lower/upper/technical school or college/university) was used as proxy variables for adult and early life socioeconomic status [12, 13].

### Early life disadvantage

Early life disadvantage was defined as reporting maternal smoking in childhood, having had severe respiratory infection before 5 years of age, being born during winter months (December, January, February), and/or reporting maternal age at delivery above 35 years. These factors have been associated with accelerated lung function decline in a previous analysis (Dratva et al., 2014, manuscript in preparation). Participants reporting any of these factors were defined as having early life disadvantage.

### Statistical analyses

Descriptive analyses of the study population and by cleaner-status were performed calculating prevalence of outcomes, exposure and covariates, using Pearson´s chi-squared test. Multiple logistic regression analysis was used to evaluate possible associations between occupational cleaning and respiratory health. Never having worked as a cleaner was used as a reference group. Associations are reported as odd ratios [OR] with 95% confidence intervals [CI]. Due to varying numbers of missing for each variable, numbers at risk differ slightly between different outcomes. Based on prior knowledge and literature, adjustments were made for age, sex, smoking habits, educational level, parents’ educational level, BMI (continuous) and participating centre. Sensitivity analyses in men, women, never smokers, current smokers, normal weight and overweight were performed. Potential heterogeneity between centres was analysed using meta-analysis according to derSimonian and Laird [14]. Analyses of associations between occupational cleaning ≥4 years and respiratory health outcomes were stratified by the dichotomous variable early life disadvantage. Potential interaction between early life disadvantages and cleaning status was analysed by including an interaction term of occupational cleaning ≥4 years and early life disadvantage in effects on respiratory outcomes. STATA (StataCorp, College Station, TX, USA), version IC 13.1, was used in all the statistical analyses.

## Results

Overall, 2138 persons (16%) reported ever having worked as a cleaner, the median duration was 2.0 years and the mean duration was 5.7 years. The highest prevalence of participants having worked as a cleaner was found in Reykjavik (21%) and Aarhus (19%); whereas reporting work as a cleaner was least common in Bergen (13%) and Uppsala (12%) (p <0.05) (Table 1).

**Table 1:** Characteristics of the study population by study centre.

	All	Aarhus	Reykjavik	Bergen	Gothenburg	Umeaa	Uppsala	Tartu
	(N = 13499)	(n = 2351)	(n = 1937)	(n = 2364)	(n = 1705)	(n = 1927)	(n = 1926)	(n = 1289)
	%	%	%	%	%	%	%	%
Men	47.0	47.2	45.5	50.1	46.9	47.3	47.6	41.9
Women	53.0	52.8	54.5	49.9	53.1	52.7	52.4	58.1
Age (years)								
Mean±SD	51.5 ±7.2	50.1 ±7.0	53.1 ±7.0	50.7 ±6.9	52.3 ±7.3	52.8 ±7.4	52.6 ±7.3	49.1 ±7.0
Smoking								
Current	17.9	19.4	18.4	24.2	17.2	11.7	11.0	25.1
Ex	46.6	51.3	59.3	57.9	40.6	35.2	34.8	38.9
Education level								
Lower	11.4	7.5	18.1	9,2	17.3	13.0	9.8	4.5
Intermediate	41.7	38.7	40.3	43.0	47.9	44.7	32.9	47.7
Higher	46.9	53.8	41.6	47.9	34.8	42.3	57.3	47.8
Occupational cleaning								
Yes	16.1	19.4	21.3	13.3	16.4	14.9	12.1	15.0
Years in occupational cleaning								
Median	2	2	2	2	3	3	3	2.5
Mean	5.7	3.5	5.6	5.3	7.2	7.8	6.5	4.2

The majority of the cleaners was female (74%), the current mean age among ever cleaners was 50.2 ±7.0 years and mean duration of work as a cleaner was 5.7 ±7.8 years. More cleaners reported current (25%) or previous smoking (51%) than those never having worked as a cleaner (16.4% and 46% respectively) (p<0.05) (Table 2). The prevalence of smoking increased with increasing duration of years having worked as a cleaner (Table 2).

**Table 2:** Characteristics of occupational cleaners in 13283 persons from Northern European population samples.

	Occupational cleaning no (n = 11145)	Occupational cleaning yes (n = 2138)		Occupational cleaning ≤ 1 year (n = 687)	Occupational cleaning >1 to <4 years (n = 566)	Occupational cleaning ≥ 4 years (n = 772)	
			p-value*				p-value*
	%	%		%	%	%	
Men	51.1	26.0	<0.001	31.6	24.9	22.8	<0.001
Women	48.9	74.0	<0.001	68.4	75.1	77.2	<0.001
Age (years)							
Mean ±SD	51.8 ±7.2	50.2 ±7.0		48.7 ±6.6	48.7 ±6.6	52.4 ±7.1	
BMI							
18.5–≤25	44.9	45.6	0.075	52.7	43.9	40.0	<0.001
> 25	54.3	53.0	0.075	46.1	54.8	58.9	<0.001
Smoker							
Current	16.4	24.5	<0.001	17.9	23.6	31.6	<0.001
Ex	45.7	51.3	<0.001	46.2	56.1	53.0	<0.001
Education level							
Lower	10.0	17.5	<0.001	7.2	11.7	30.1	<0.001
Intermediate	41.4	43.0	<0.001	35.8	45.7	48.1	<0.001
Higher	48.6	39.6	<0.001	57.0	42.6	21.9	<0.001
Mother´s education							
Lower	52.3	51.0	<0.001	45.6	49.8	57.9	<0.001
Intermediate	22.5	22.5	<0.001	25.2	22.8	18.8	<0.001
Higher	9.1	7.2	<0.001	10.8	7.6	3.8	<0.001
Father´s education							
Lower	43.4	41.4	<0.001	36.1	41.7	47.1	<0.001
Intermediate	25.7	27.1	<0.001	28.7	25.3	26.1	<0.001
Higher	14.7	11.9	<0.001	16.9	12.7	6.7	<0.001
Early life disadvantage†	58.6	62.2	0.002	63.0	62.7	61.4	0.015
Maternal age >35 years	13.3	12.3	0.26	12.3	13.3	11.4	0.54
Born during winter months	23.9	22.8	0.28	21.9	20.7	25.8	0.11
Childhood respiratory infection	6.6	8.5	<0.001	8.6	9.3	7.4	<0.001
Maternal smoking	33.8	39.3	<0.001	40.3	40.4	37.7	<0.001
Wheeze last 12 months	18.0	25.5	<0.001	20.3	25.0	29.9	<0.001
Asthma symptoms‡	13.0	20.8	<0.001	14.6	20.7	26.2	<0.001
Adult onset asthma§	6.3	9.3	<0.001	6.7	9.9	11.7	<0.001
Current asthma medication	7.1	10.2	<0.001	6.3	11.0	12.8	<0.001
Self-reported COPD	2.4	5.0	<0.001	3.2	5.1	6.0	<0.001
Chronic bronchitis	8.4	12.6	<0.001	8.6	12.7	16.3	<0.001

* p-values from Pearson´s chi-squared test.

† Any of the following: Born during winter months (December, January, February), maternal age >35 years, severe respiratory infection before the age of 5 years and/or maternal smoking.

‡ Asthma symptoms: Yes to three or more out of 6 questions on asthma-like symptoms.

§ Asthma after 16 years of age.

Respiratory symptoms, asthma, self-reported COPD and chronic bronchitis were significantly more prevalent among ever cleaners (p<0.001) (Table 2). The occurrences of wheeze in the last 12 months, asthma symptoms, adult onset asthma, current use of asthma medication, self-reported COPD, and chronic bronchitis were all significantly increased with time worked as a cleaner (p<0.001) (Table 2).

**Fig 1:**
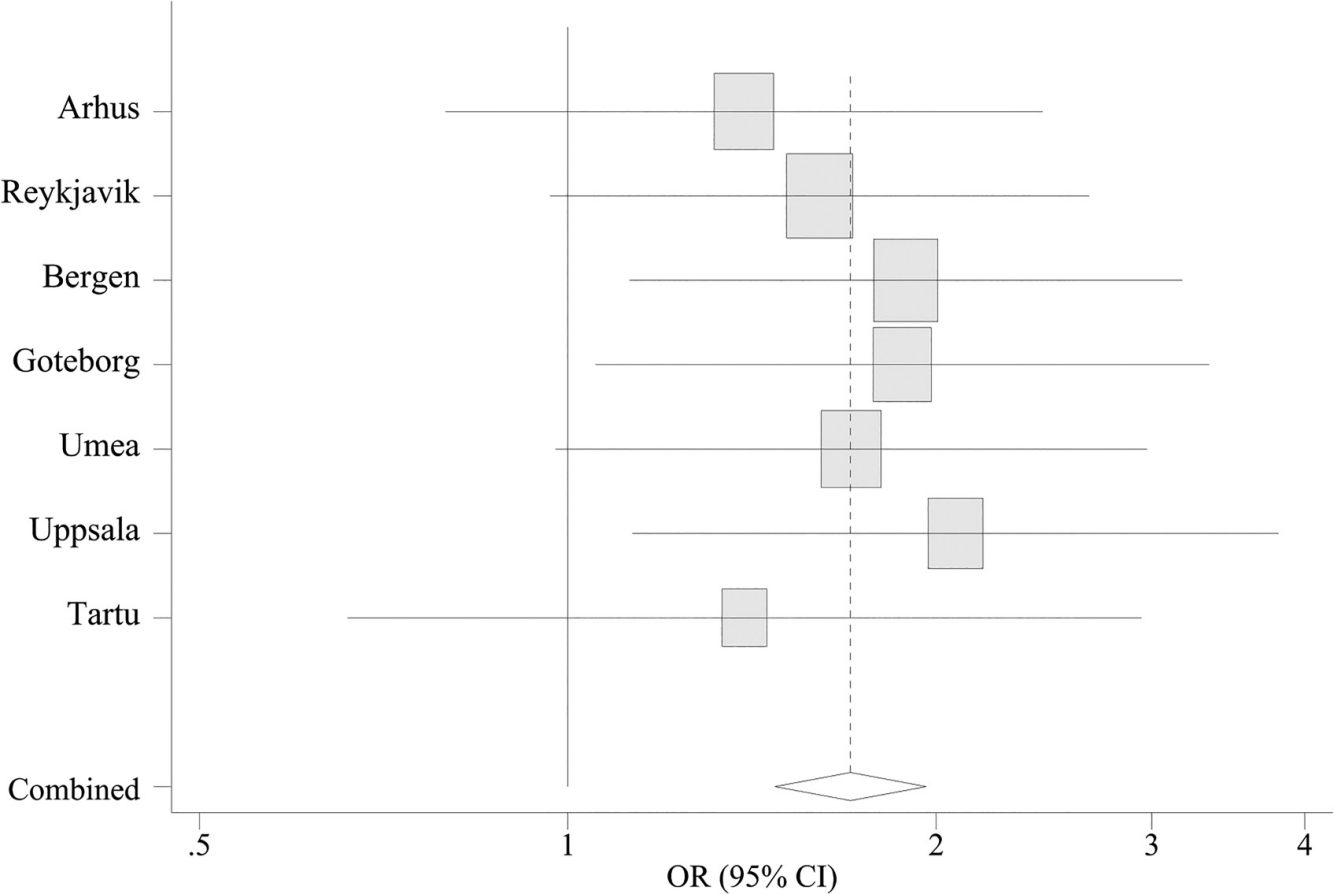
Adjusted odd ratios for the association of occupational cleaning with asthma symptoms (≥3 symptoms) in each study centre, as estimated from meta-analysis. For each centre the square gives the OR and the horizontal lines indicate 95% CI; the area of the square is proportional to the size of the study sample in each centre. Adjustment within each centre made for sex, smoking habits, age, education level, parent´s education level, BMI and participating centre. For combined odds ratios, the diamond indicates 95% CI from a model with centre as random effect. p-value for heterogeneity = 0.8.

There was a significantly increased risk for self-reported COPD among cleaners (OR 1.69 [95% CI 1.29–2.20]), as well as for respiratory symptoms (1.44 [1.27–1.62]; 1.66 [1.46–1.90]) and asthma (1.47 [1.22–1.77]), after adjusting for potential confounding factors (Table 3). Those having worked with cleaning for more than one year had a significantly increased risk of wheeze last 12 months (1.46 [1.18–1.83]; 1.62 [1.34–1.96]), asthma symptoms (1.68 [1.33–2.31]; 2.04 [1.67–2.49]), current use of asthma medication (1.65 [1.23–2.21]; 1.67 [1.30–2.15]), self-reported COPD (1.80 [1.14–2.85]; 1.65 [1.14–2.42]) and chronic bronchitis (1.77 [1.34–2.32]; 1.84 [1.46–2.32]) compared to those never having worked as a cleaner. The risk for asthma, bronchitis and respiratory symptoms increased with increasing time having worked as a cleaner (Table 3). There was no significant heterogeneity between the participating centres with respect to respiratory symptoms and disease; the results for three or more asthma symptoms are presented in Fig 1.

**Table 3:** Respiratory health outcomes as associated with occupational cleaning in 13283 persons from Northern Europe population samples.

	**Occupational cleaning yes (n = 2138)**		**Occupational cleaning ≤1 year (n = 687)**	**Occupational cleaning >1 to <4 years (n = 566)**	**Occupational cleaning ≥ 4 years (n = 772)**	
	Adj. OR*		Adj. OR*	Adj. OR*	Adj. OR*	
	(95% CI)	p- value	(95% CI)	(95% CI)	(95% CI)	||p for trend
Wheeze last 12 months	1.44 (1.27–1.62)	<0.001	1.22 (0.98–1.52)	1.46 (1.18–1.83)	1.62 (1.34–1.96)	<0.001
Asthma symptoms†	1.66 (1.46–1.90)	<0.001	1.20 (0.94–1.54)	1.68 (1.33–2.31)	2.04 (1.67–2.49)	<0.001
Adult onset asthma‡	1.47 (1.22–1.77)	<0.001	0.92 (0.65–1.31)	1.44 (1.05–1.97)	1.59 (1.22–2.08)	<0.001
Current asthma medication	1.46 (1.23–1.73)	<0.001	0.85 (0.60–1.21)	1.65 (1.23–2.21)	1.67 (1.30–2.15)	<0.001
Self-reported COPD	1.69 (1.29–2.20)	<0.001	1.41 (0.85–2.33)	1.80 (1.14–2.85)	1.65 (1.14–2.42)	<0.001
Chronic bronchitis	1.45 (1.24–1.70)	<0.001	1.04 (0.77–1.43)	1.77 (1.34–2.32)	1.84 (1.46–2.32)	<0.001

* Adjusted for sex, age, smoking, education level, parents’ education level, BMI and participating centre.

† Asthma symptoms: Yes to three or more out of 6 questions on asthma-like symptoms.

‡ Asthma after 16 years of age.

|| p for trend was estimated using years of exposure as a continuous variable.

Increased risk of wheeze, three or more asthma symptoms and chronic bronchitis among occupational cleaners was consistently found in sensitivity analysis of various subgroups (Table 4).

**Table 4:** Sensitivity analysis: The association of occupational cleaning with respiratory symptoms in various subgroups.

	Occupational cleaning yes		
	Wheeze	Asthma symptoms*	Chronic bronchitis
	Adj. OR† (95% CI)	Adj. OR† (95% CI)	Adj. OR† (95% CI)
Men (n = 555)	1.49 (1.19–1.87)	1.72 (1.34–2.20)	1.60 (1.22–2.11)
Women (n = 1582)	1.55 (1.32–1.81)	1.73 (1.46–2.06)	1.59 (1.29–1.95)
Never smoker (n = 831)	1.42 (1.15–1.77)	1.53 (1.21–1.94)	1.33 (0.99–1.77)
Current smoker (n = 509)	1.38 (1.11–1.72)	1.53 (1.20–1.95)	1.41 (1.07–1.87)
Normal weight (BMI 18.5–≤25) (n = 949)	1.35 (1.10–1.65)	1.63 (1.31–2.04)	1.78 (1.39–2.28)
Overweight (BMI>25) (n = 1104)	1.57 (1.33–1.85)	1.68 (1.41–2.01)	1.39 (1.11–1.73)

* Asthma symptoms: Yes to three or more out of 6 questions on asthma-like symptoms.

† Adjusted for sex, age, smoking, education, parents’ education and participating centre.

**Fig 2:**
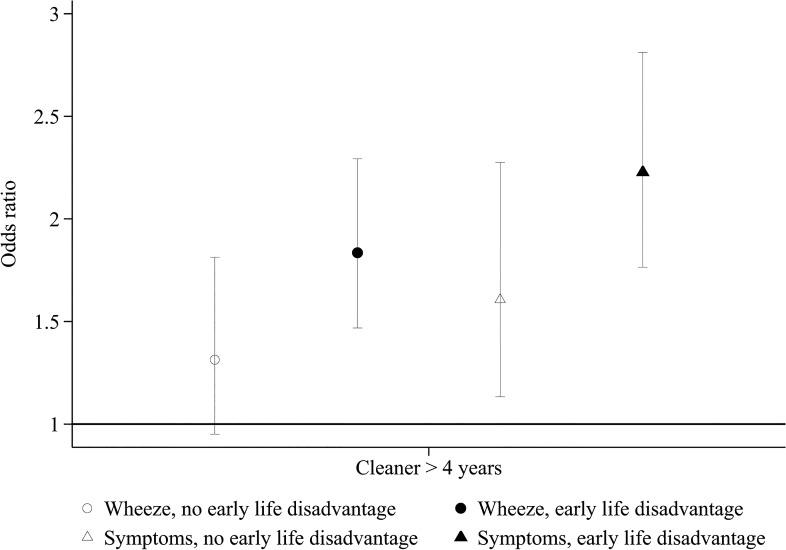
Associations of occupational cleaning ≥4 years with respiratory symptoms in persons with and without early life disadvantage.

Respiratory symptoms and diseases were more common in cleaners, both among persons with early life disadvantage and among those without such disadvantage. However, the asthma risk associated with occupational cleaning generally appeared to be higher among those with early life disadvantage (Table 5) (Fig 2). Interaction between early life disadvantage and occupational cleaning was statistically significant for the most prevalent outcome, wheeze (p_interaction_ 0.035), and p_interaction_ was 0.053 for ≥3 asthma symptoms (Table 5). A similar tendency was not indicated for chronic bronchitis and self-reported COPD. S1 Table shows analysis of the association of occupational cleaning with wheeze stratified by each of the components of the “early life disadvantage” factor.

**Table 5:** Association of occupational cleaning with respiratory outcomes in persons with and without early life disadvantage.

	No early life disadvantage*			Early life disadvantage*			
	Occupational cleaning no	Occupational cleaning ≥ 4 years		Occupational cleaning no	Occupational cleaning ≥ 4 years		
	(n = 4617)	(n = 298)		(n = 6524)	(n = 474)		
			Adj. OR†			Adj. OR†	p_interaction_ ‡
	%	%	(95% CI)	%	%	(95% CI)	
Wheeze last 12 months	16.2	22.6	1.32 (0.96–1.82)	19.3	34.5	1.83 (1.47–2.29)	0.035
Asthma symptoms§	11.4	19.2	1.61 (1.13–2.28)	14.0	30.4	2.23 (1.76–2.81)	0.053
Adult onset asthma||	5.9	9.9	1.54 (0.98–2.43)	6.6	12.9	1.72 (1.25–2.35)	0.48
Current asthma medication	6.1	11.6	1.77 (1.17–2.68)	7.8	13.5	1.60 (1.18–2.16)	0.74
Chronic bronchitis	8.3	14.8	1.79 (1.23–2.60)	8.5	17.3	1.94 (1.47–2.57)	0.43

* Any of the following: Born during winter months (December, January, February), maternal age >35 years, severe respiratory infection before the age of 5 years, maternal smoking.

† Adjusted for sex, age, smoking, education level and participating centre.

‡ Test for interaction between 4-year+ occupational cleaner and early life disadvantage.

§ Asthma symptoms: Yes to three or more out of 6 questions on asthma-like symptoms.

|| Asthma after 16 years of age.

## Discussion

This analysis observed that persons who had worked as cleaners had increased risk of respiratory symptoms, asthma and self-reported COPD. The suggested association with self-reported COPD was supported by increased risk also for symptoms of chronic bronchitis. For most outcomes, there was a trend toward a dose-response relationship with higher risks for those having worked as cleaners for longer duration. The findings were consistent across study centres from different countries in Northern Europe. To our knowledge, this analysis shows the first evidence of early life influence on susceptibility to an occupational hazard: Associations between occupational cleaning and respiratory symptoms were stronger among persons with early life disadvantage; thus, early life disadvantage appeared to constitute an important effect modifier by increasing the vulnerability to the harmful effects of occupational cleaning exposures. Our analyses suggest that early life disadvantage may increase susceptibility to chemical exposures later in life, a concept that may have considerable impact on public health policies.

An important novel finding of this paper regards the observed effect modification of exposure to cleaning agents by early life disadvantage factors. The “early life disadvantage” factor in this analysis was based on biological plausibility and previous knowledge [15, 16]; thus, this factor conveys a general concept beyond the individual components. An interaction between working as a cleaner and early life disadvantage was significant for wheeze and borderline significant for asthma symptoms. The findings are supported by two other analyses showing how factors reflecting early life disadvantage increased the susceptibility to the harmful effects from personal smoking with regard to airflow limitation [4] and lung function decline (Dratva et al., manuscript in preparation). A previous study showed that individuals with early life disadvantage were at risk of accelerated lung function decline and had substantially increased risk for COPD [3]. One may speculate that early life disadvantage might impair molecular maintenance mechanisms, and thereby reduce the ability to later in life deal with subsequent insults from harmful exposures such as cleaning chemicals.

This analysis confirmed the increased health hazard from work as a cleaner seen in several previous studies with similar increased risk of airway symptoms and asthma [17, 18, 19, 20, 21]. Proposed mechanisms include irritant effects as well as potential specific immunological effects. Although occupational exposures and COPD is described for other vocations [22, 23], there is little literature concerning COPD in occupational cleaners. An association seems biological plausible, given emerging understanding of inflammatory effects from cleaning chemicals [21] as well as potential for chronification of asthma on exposure after symptom debut. Repeated inhalation of irritants from cleaning chemicals, either from daily low-dose exposure or multiple single high-dose exposures, can cause damage to the bronchial epithelium through a pro-inflammatory response, neurogenic inflammation due to exposed nerve endings and increased lung permeability, and remodelling of the airway epithelium [24, 25, 26].

The strengths of this study include its population-based design, the extensive data from the participants, the large number of participants and the multicentre structure. That the participants were randomly selected from a general population makes the results applicable to a general population rather than to select groups. Furthermore, the data from the participants are reasonably extensive, ensuring that each individual is well characterised. Because of the large number of participants, the study had enough power to investigate possible interactions between work as a cleaner and early life disadvantage.

There was no apparent heterogeneity between the different centres, supporting an interpretation of the results in terms of biological mechanisms rather than confounding by sociocultural factors that are likely to differ between centres.

This analysis has some methodological challenges. Asthma and COPD were defined by self-report and could be subject to misclassification bias. Differential misclassification bias with regard to occupational cleaning is possible and could cause positive or negative confounding. However, the findings were generally consistent with results for symptoms of asthma or chronic bronchitis, for which non-differential misclassification is more likely than differential misclassification. Further, the findings were robust for various adjustments and consistent between subgroups and study centres. Reporting error in cleaning exposure assessment is likewise more likely to give non-differential bias. An analysis of adult reporting of childhood factors found high repeatability and that the reporting error was non-differential with regard to asthma and asthma symptoms [27]. It seems unlikely that error in reporting early life factors could have created the effect modification observed in the present study. We believe the overall result of misclassification in the present analysis may have led to underestimation of true effects.

The definition of COPD was based on self-reported doctor’s diagnosed COPD, a definition that gives the lowest estimates of prevalence [28, 29], as COPD is substantially under diagnosed [30]. The numbers with self-reported diagnosed COPD in the study population where small, also due to the relatively young age group studied. Low numbers might explain less consistent findings on analyses of self-reported COPD in subgroups. Residual confounding by smoking is likely, thus, sensitivity analyses of never-smokers were conducted. This gave similar results as in the whole population, suggesting that the observed results were not related to rest confounding by smoking. As data on occupational cleaning was only collected in the last RHINE survey and with limited detail, it was not possible to confirm a temporal relationship between starting work as a professional cleaner and the development of asthma and COPD. Further, our data did not allow for more detailed exploration of susceptibility windows in adulthood.

The exposure assessment in the present paper (“having worked as a cleaner”) is very crude. It is likely that occupational groups exposed to cleaning agents such as industrial cleaners and homecare takers [31] may be included in the reference category. The reference group further included occupational groups with known asthma risk [32, 33], thereby leading to an underestimation of the respiratory health risk related to occupational cleaning. It is possible that symptomatic subjects more often would quit work as cleaner. This potential “healthy worker effect” may further contribute to an underestimation of the negative impact from cleaning work [34] and might explain why there was not a stronger trend with increasing years in cleaning occupation. Overall, while the analysis have several methodological challenges; these are likely to have attenuated the associations and cannot easily explain an interaction between early life disadvantage and occupational exposure on asthma symptoms.

In conclusion, this analysis found increased asthma, respiratory symptoms and self-reported COPD in persons who have worked as occupational cleaners, particularly among persons with early life disadvantage. The findings suggest that early life disadvantage might increase individual susceptibility to an adult occupational exposure. This seems biologically plausible, and agrees with findings of increased susceptibility to smoking in persons with factors included in early life disadvantage, such as maternal smoking. This finding needs further confirmation in other studies, as there may be widespread implications. Firstly, this may lead to focused mechanistic research as to how early life disadvantage may influence adult respiratory health, and how responses to a chemical hazard may be modified. Secondly, work-place interventions might focus in particular on persons with early life disadvantage, although optimal work-place air quality and adequate protective measures are important for all. Finally, long-term respiratory health effects of exposures in cleaning occupations need further investigation, as this study indicated increased risk of self-reported COPD in cleaners. Given the large scale use of cleaning agents and disinfectants in occupational setting as well as in private homes, it is essential to understand modifying factors, as well as long-term and short-term respiratory health consequences of such exposures.

## Supporting Information

S1 Table Association of occupational cleaning with respiratory symptoms in subgroups according to each of the components of the “early life disadvantage” factor. * Adjusted for age, smoking, education level and participating centre.
(DOCX)
