# Supplementary material for: Respiratory Health in Cleaners in Northern Europe: Is Susceptibility Established in Early Life?
Source: PLoS One. 2015 Jul 13;10(7):e0131959. doi: 10.1371/journal.pone.0131959 (PMC4500550; doi:10.1371/journal.pone.0131959)
Supplement: S1 Table — * Adjusted for age, smoking, education level and participating centre. (DOCX) [file pone.0131959.s001.docx]

**S1 table.** Association of occupational cleaning with respiratory symptoms in subgroups according to each of the components of the “early life disadvantage” factor.

|  | **Occupational cleaning ≥4 years (n=772)** |
| --- | --- |
|  | **Wheeze** |
|  | Adj. OR* (95% CI) |
| Maternal age >35 years |  |
| No | 1.67 (1.38-2.02) |
| Yes | 1.37 (0.74-2.55) |
| Born during winter months |  |
| No | 1.55 (1.25-1.92) |
| Yes | 1.99 (1.40-2.82) |
| Childhood respiratory infection |  |
| No | 1.72 (1.42-2.08) |
| Yes | 1.10 (0.58-2.11) |
| Maternal smoking |  |
| No | 1.53 (1.20-1.95) |
| Yes | 1.85 (1.39-2.45) |

* Adjusted for age, smoking, education level and participating centre.
